# Improving the Concrete Crack Detection Process via a Hybrid Visual Transformer Algorithm

**DOI:** 10.3390/s24103247

**Published:** 2024-05-20

**Authors:** Mohammad Shahin, F. Frank Chen, Mazdak Maghanaki, Ali Hosseinzadeh, Neda Zand, Hamid Khodadadi Koodiani

**Affiliations:** 1 Mechanical Engineering Department, The University of Texas at San Antonio, San Antonio, TX 78249, USA; mohammad.shahin@utsa.edu (M.S.); ali.hosseinzadehghobadlou@utsa.edu (A.H.); 2 Computer Science Department, The University of Texas at San Antonio, San Antonio, TX 78249, USA; 3 Civil & Environmental Engineering Department, The University of Texas at San Antonio, San Antonio, TX 78249, USA

**Keywords:** maintenance, inspection, concrete crack detection, big data, waste reduction, machine learning, Industry 4.0, computer-based vision

## Abstract

Inspections of concrete bridges across the United States represent a significant commitment of resources, given their biannual mandate for many structures. With a notable number of aging bridges, there is an imperative need to enhance the efficiency of these inspections. This study harnessed the power of computer vision to streamline the inspection process. Our experiment examined the efficacy of a state-of-the-art Visual Transformer (ViT) model combined with distinct image enhancement detector algorithms. We benchmarked against a deep learning Convolutional Neural Network (CNN) model. These models were applied to over 20,000 high-quality images from the Concrete Images for Classification dataset. Traditional crack detection methods often fall short due to their heavy reliance on time and resources. This research pioneers bridge inspection by integrating ViT with diverse image enhancement detectors, significantly improving concrete crack detection accuracy. Notably, a custom-built CNN achieves over 99% accuracy with substantially lower training time than ViT, making it an efficient solution for enhancing safety and resource conservation in infrastructure management. These advancements enhance safety by enabling reliable detection and timely maintenance, but they also align with Industry 4.0 objectives, automating manual inspections, reducing costs, and advancing technological integration in public infrastructure management.

## 1. Introduction

Industry 4.0 (I4.0) brings a multitude of technological advancements like Artificial Intelligence (AI), robotics, drones, and computer vision, each with a unique contribution towards improving crack detection. Through computer vision and Machine Learning (ML) algorithms, AI sifts through vast historical and real-time datasets to identify potential cracks early on, paving the way for timely preventative maintenance. Drones, outfitted with high-resolution cameras, offer the advantage of remote inspections and real-time monitoring of large concrete structures, significantly cutting down on manual inspection needs while bolstering safety measures. Computer vision emerges as a cornerstone for automated crack detection, scrutinizing images and video feeds to spot anomalies such as cracks with precision. Beyond their standalone capabilities, integrating these technologies creates a collaborative ecosystem conducive to data fusion. This integrated setup accelerates real-time decision-making and facilitates prompt corrective actions upon crack detection. This blend of AI and I4.0 technological innovations elevates the accuracy and efficiency of concrete crack detection. It heralds a proactive approach towards Inspection 4.0. 

In addition to drawing on this integration, the paper compares multiple computer vision models to benchmark them against each other on a single dataset. Comparing different computer vision models on a single dataset is a practice of substantial significance, offering a fair and consistent platform for performance evaluation. This approach ensures unbiased comparisons and generates comparable performance metrics, which are crucial in assessing the robustness of different algorithms in handling challenges like noise, occlusion, and variations in lighting and scale. Identifying the strengths and weaknesses of each model becomes feasible, which is instrumental in selecting the suitable model for a particular application. Moreover, it aids in establishing standardized benchmarks, crucial for gauging progress over time and against the state-of-the-art, thereby fostering a competitive environment for algorithm improvement. Insights into the generalization capabilities of different models across varying data conditions within the same dataset are gleaned, which is pivotal for real-world applications.

Additionally, this comparison provides a vantage point to optimize computational resources, as different models may have varying computational and memory requirements. This comparison practice on a standard dataset validates findings and advances the field by promoting transparency and reproducibility in research. Furthermore, it sheds light on the importance of different features for the task at hand, thus aiding in feature engineering and model refinement.

According to the American Society of Civil Engineering (ASCE), one in every nine bridges is subjected to collapse at any given moment in the United States. Federal guidelines require inspections every year. Currently, there are more than 600,000 bridges across the United States. This routine process consumes time, money, labor, and materials. Understanding the steps taken to ensure the completion of the process is essential for process improvement [1,2].

**Figure 1:**
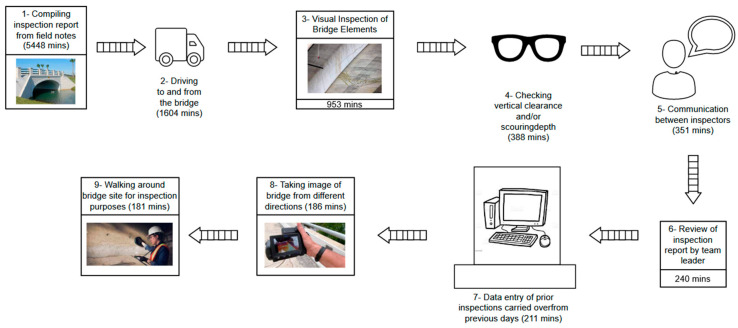
VSM for the routine bridge inspection process based on data from the American Society of Civil Engineers 2020.

Different strategies were devised for scheduling inspections of fracture-prone bridges, as described in multiple studies [3,4,5]. Also, Madanat et al. [6] designed and developed digital tools for decision-making that aid in selecting appropriate corrective actions and allocating resources for bridge maintenance, thereby enabling quicker scanning of more bridges [7]. Recently, the bridge inspection process has begun incorporating I4.0 technologies [8], including drones [9,10] photogrammetry [11,12,13], virtual reality [12,14], and database management systems. A significant innovation has been the implementation of time-dependent reliability analysis, which leverages historical data from visual inspections to predict future structural performance [15,16]. Table 1 shows a summary of improvements suggested or applied to each category. Figure 1 shows the bridge inspection process’s Value Stream Mapping (VSM). The details of the inspection process were collected by Clarke-Sather et al. [2].

Bridge inspections are crucial for several reasons, including ensuring public safety, maintaining structural integrity, identifying repair and maintenance needs, prolonging the bridge’s lifespan, and facilitating efficient resource allocation [17,18,19,20]. Regular inspections help identify and address potential issues before they become critical, ensuring the safety of all bridge users, prolonging the lifespan, and enabling efficient resource allocation for maintenance and repairs [21]. Table 2 summarizes some key reasons why concrete inspection in the structure of a bridge is important.

This paper begins with an Introduction, presenting the importance of crack detection, manual detection limitations, and AI’s role in inspection. It then covers Structural Health Monitoring (SHM) and AI in SHM. Next, it discusses Section 2 Inspection 4.0, followed by the Section 3 Dataset. The Section 4 follows, detailing the algorithms used. Then, Section 5 analyze the findings, followed by Section 6. Finally, the Section 7 wraps up the study.

### 1.1. Structural Health Monitoring Systems

The SHM system is a critical practice dedicated to continually assessing the integrity of civilian infrastructure. The primary objective of SHM is to monitor a structure’s current health status compared to its baseline state, identify any deviations or damages, and develop appropriate maintenance strategies to address these issues [22]. This section will explore the various methods available for evaluating structural health, employing various tools [23]. Damage detection within SHM typically involves identifying changes that alter the physical properties of a structure, thereby compromising its integrity. Such damage often manifests as cracks, considered significant indicators of potential structural failure.

SHM can include tilt sensors, optical displacement sensors, and wireless platforms designed to monitor structural health and environmental conditions. Combined with AI algorithms, these sensors help reduce costs, minimize maintenance, and enhance safety by providing real-time data and alerts. Tilt sensors measure the inclination, or angle, of an object with respect to gravity. They are crucial for monitoring structural movements and deformations, especially in buildings, bridges, and other infrastructure, ensuring that deviations from the norm are detected early. The optical displacement sensors track the distance changes between a sensor and a target object using light, typically lasers. They are highly accurate and used for monitoring the minute movements in structures, which can indicate stress, potential failure, or a need for maintenance. These sensors and other platforms integrate into a network that can communicate data wirelessly, facilitating real-time monitoring of structural health and environmental conditions. This setup allows continuous surveillance without the need for physical data retrieval, improving safety and efficiency [24].

Recent technological advancements have revolutionized the SHM field by integrating data-centric technologies to enhance the safety of civil infrastructure. The last two decades have seen SHM evolve significantly, driven by intelligent, mobile sensor systems. Concurrently, smartphones have emerged as pivotal tools in SHM by facilitating innovative applications through intelligent, distributed, and participatory sensor networks. This section elaborates on the role of smartphones in SHM. It explores how public participation can be incorporated into SHM frameworks. Unlike traditional methods, these modern approaches sometimes suffer from variable control over sensor operations, such as timing and placement [25,26]. These variances, termed citizen-induced uncertainties, are addressed by proposing multisensory solutions centered around smartphones, enabling real-time updating of civil infrastructure models, such as bridges, using data collected from the public.

Understanding and addressing these flaws is crucial for ensuring the safety and longevity of critical infrastructure, including roads, subway systems, bridges, buildings, dams, tunnels, and landmarks. This section aims to clarify how SHM systems can effectively identify and mitigate these risks to maintain structural health and safety.

#### 1.1.1. Cracks

**Figure 2:**
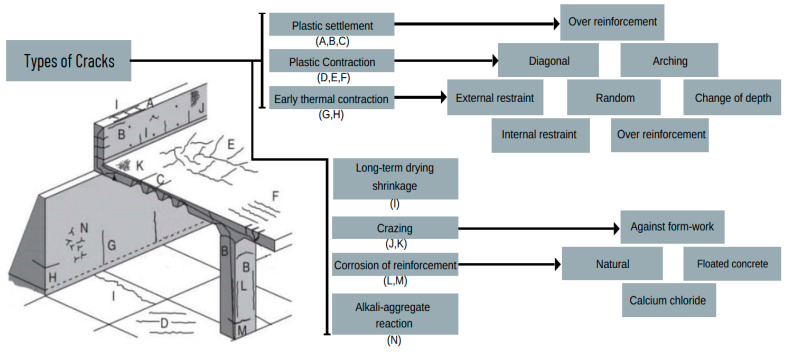
Classification of concrete cracks.

Regarding imagery, cracks represent abrupt shifts in pixel brightness. Cracks manifest as slender, dark streaks on a solid surface, indicating where the material has divided without separating. Various factors can lead to the emergence of cracks on a concrete surface: fluctuations in material dimensions, foundational movements, early drying, undue weight, water-induced stress, uneven mixtures, expanding soil, inadequate soil support, wear over time, settling, and other activities. Concrete expands and matures with moisture or temperature shifts, like many building materials. Additionally, the weight it bears, whether from its mass or other loads or its foundational support, can cause it to bend or deflect. If there are not adequate measures to allow for these natural movements, this can result in cracks in the concrete. In Figure 2, various types of cracks are depicted, including those caused by plastic shrinkage, improper jointing, continuous external restraint, absence of isolation joints, freeze–thaw cycles, crazing (also known as craze cracks), and settlement-related cracking. While some cracks are easily discernible as imperfections, others can evolve into significant hazards, even if they are initially minor. The intricate background patterns can obscure inevitable cracks, making them hard to detect. Cracks exhibit varying widths: hairline cracks, measuring 0.1 mm across, are noticeable against simple backdrops but can be tricky to spot under changing lighting. Fine cracks span up to 1 mm, while cracks with widths up to 5 mm are typically not deemed harmful and can be mended. Conversely, cracks exceeding a 5 mm width can lead to substantial harm and might necessitate extensive repairs or even replacements [27].

**Figure 3:**
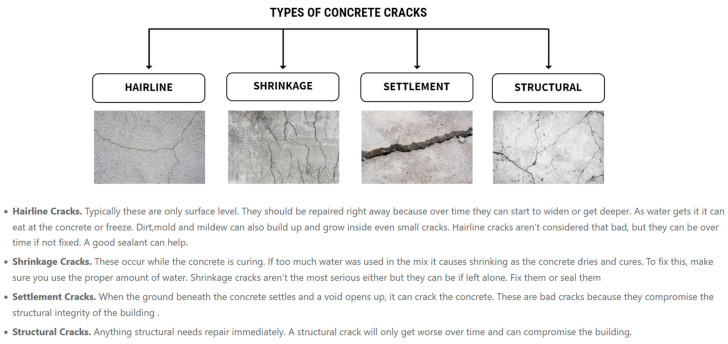
Types of concrete cracks.

Cracks emerging during the initial stages of the concrete setting are not a significant concern, as they do not compromise the structure’s durability, stability, or lifespan. However, if left unrepaired, they can pose a considerable risk during the structure’s life span. In addition, cracks that develop later on pose a more substantial threat to the structural safety and longevity of the concrete structures [27]. Examples include those resulting from freeze–thaw cycles or settling. Such defects can lead to the degradation of the concrete structure. Most often, these flaws arise from suboptimal design and building techniques, such as damaged joint placement, lacking necessary isolation and building joints, poor groundwork or soil preparation, over-watering the concrete mix or using overly fluid mixtures, mishandling of concrete finishing, and insufficient or incorrect curing methods. While it is not feasible to entirely prevent concrete from cracking, adopting sound construction methods can significantly reduce the likelihood of such issues. Regarding final appearance, these cracks can be classified into the four main types shown in Figure 3.

#### 1.1.2. Manual Crack Detection

Identifying cracks involves pinpointing or discerning signs of cracks in structures through expert human intervention or technological means. The endeavor to spot telltale signs, such as cracks, on structures like roads, subway systems, bridges, buildings, dams, landmarks, and more relies on an amalgamation of techniques. Cracks often serve as early warnings, signifying structural deterioration. Spotting these cracks is pivotal for upkeep and demands meticulous oversight. Regular evaluations are imperative to assess structural health, with timely crack identification potentially averting more severe issues. This is vital for ensuring public safety and protecting the structure’s longevity. Two primary approaches to crack identification exist: a manual, hands-on assessment and an automated assessment. The hands-on approach, reliant on human intervention, necessitates expertise. Equipped with the right tools, professionals meticulously examine structures while adhering to safety norms. However, this method can be expensive, labor-intensive, and time-consuming, if not sometimes hazardous. Furthermore, since it lacks a visual recording component, it demands comprehensive record-keeping, and judgments about crack severity can be challenging. Hands-on assessments can be protracted for expansive infrastructure, given the extensive areas that need coverage [28].

### 1.2. Artificial Intelligence in Structural Health Monitoring Systems

#### 1.2.1. Artificial Intelligence

**Figure 4:**
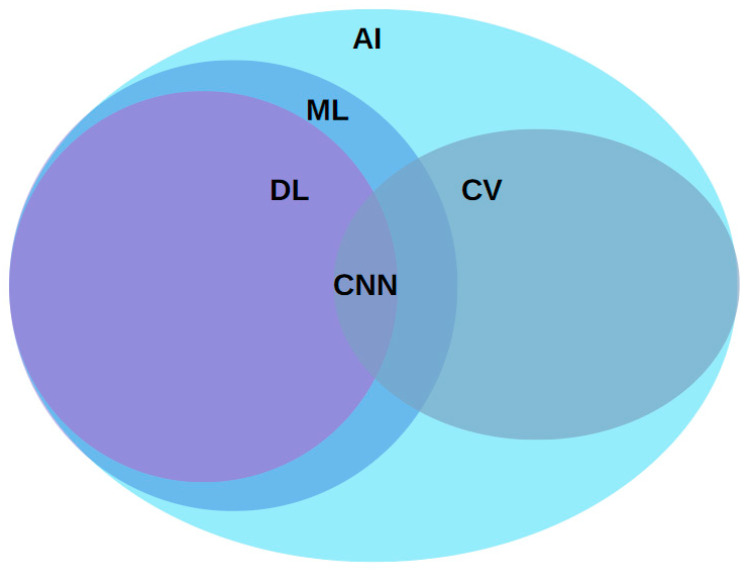
A visual explanation of the relationship between AI, ML, DL, and Computer Vision (CV).

AI, pioneered by John McCarthy in 1955, encompasses subfields like ML, Deep Learning (DL), and Neural Networks (NN). ML focuses on algorithms that learn from data to enhance performance, utilizing statistical methods [29]. DL, a branch of ML, involves Convolutional Neural Networks (CNN) that mimic the human brain to process complex data efficiently, often outperforming traditional ML techniques [30]. These technologies are applied in various areas, such as object detection, video action classification, and 3D modeling [31]. A concise overview of these interconnections is shown in Figure 4, emphasizing the relevance of these AI components to the broader scope of our study.

#### 1.2.2. Crack Attributes

Attributes, or features, are essential data points that define an object and assist systems in recognizing and classifying it. In image processing, morphological operations like erosion and dilation help analyze and enhance the differentiation of pixels, which is particularly useful in identifying crack characteristics. Machine learning utilizes designated attributes to extract details from images. At the same time, neural networks automatically process attribute data through multiple layers, each refining the extraction of characteristics like edges, patterns, and brightness, ultimately distinguishing between cracked and uncracked pixels.

#### 1.2.3. Crack Detection Using Statistical Methods

Image processing techniques for crack detection involve several key steps, including image pre-processing, segmentation, feature extraction, and crack identification. These methods utilize edge detection to identify abrupt pixel intensity transitions and segmentation to isolate objects. Various strategies, including morphological operations, statistical methods, and pattern matching, help classify cracks. Techniques also leverage contrast differences between crack pixels and their surroundings, applying mathematical morphology and curvature evaluations to enhance crack visibility and segmentation [32,33,34,35,36,37,38,39,40,41,42,43].

Ensuring structural integrity requires cost-effective, automated crack detection methods. However, image processing for crack detection is complex and influenced by environmental factors such as shadows, lighting conditions, and background noise [44,45,46,47,48,49,50,51]. Crack detection accuracy is also affected by the camera’s positioning and resolution. Various challenges hinder the effectiveness of contrast and intensity-based algorithms, including image orientation, lack of depth data, variability in thresholds and outcomes, and the manual identification of crack endpoints [52,53].

#### 1.2.4. Crack Detection Using Machine Learning Methods

ML has incredibly advanced crack detection, using methods like graph-cut segmentation to identify crack features [54]. Techniques such as Deep Belief Networks (DBN) differentiate and classify cracks based on geometric characteristics and color texture attributes [55,56,57,58,59,60,61]. However, these methods can struggle with obscure cracks and complex non-linear regression tasks. ML techniques generally require extensive structured labeling and often do not perform as well as deep learning methods, which better interpret nuanced features [62,63].

#### 1.2.5. Crack Detection Using Deep Learning Methods

Over the last decade, DL models, particularly CNNs, have gained prominence in computer vision due to technological advances in processing and storage [64,65]. CNNs are crucial for tasks like image classification and recognition, utilizing a feed-forward topology [66,67,68,69]. Additionally, Long Short-Term Memory (LSTM) networks, a type of Recurrent Neural Network (RNN), excel in sequence prediction by maintaining data across processing stages [70,71,72,73], often integrating with CNNs for enhanced object recognition.

**Figure 5:**
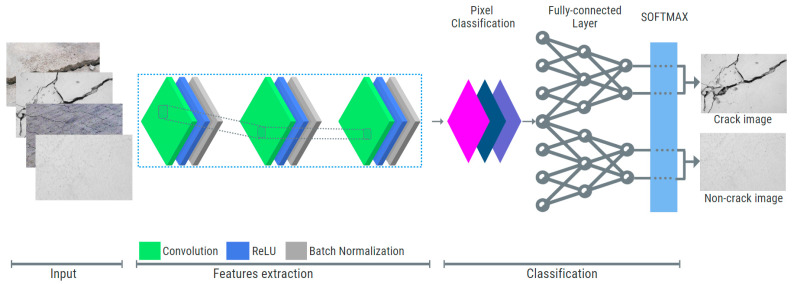
Basic blocks of the NN-based model in image classification.

Fully Convolutional Neural Networks (FCNs) are notable for their absence of fully connected layers, enhancing efficiency in image processing tasks such as fake fingerprint detection by offering high accuracy, faster processing, and reduced memory needs [74,75]. Region Proposed Networks (RPNs), including models like Faster R-CNN, are applied in object detection in diverse areas such as extracting information from receipts, recognizing handwritten text, and even identifying mathematical expressions in documents [76,77,78]. Figure 5 shows the basic architecture used in CNN.

**Figure 6:**
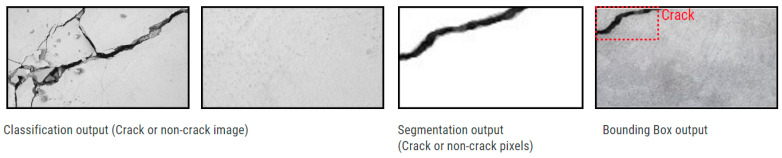
DL output illustrations.

In CNNs, initial layers detect basic patterns like edges, intermediate layers discern shapes and colors, and advanced layers capture detailed object features. After these layers extract data, it feeds into a fully connected neural network for classification or segmentation layers for more detailed analysis. CNNs, a deep forward-propagating neural network, are versatile across various data types and are used for classifying, localizing, or segmenting image cracks. See Figure 6 for output illustrations. Our paper employed crack detection through classification output.

Deep Convolutional Neural Networks (DCNNs) are effective for understanding complex correlations between inputs and outputs, aiding in data classification and segmentation. They are instrumental in crack identification, where they process high-resolution images that require significant computational resources. Visual Transformer (ViT) models, a recent advancement in DL, excel in tasks like image classification and object detection, offering robust performance and enhanced privacy features in image processing applications.

## 2. Inspection 4.0

Automation and Information Technology (IT) integration have reshaped the functions of managers, engineers, and operators, resulting in workplaces that rely more on knowledge [79]. As a result, deeper integration of automation and IT has become crucial [79]. Data science transforms vast amounts of data into actionable insights, enhancing transparency and product quality [80,81]. Utilizing sensors for quality control and applying sophisticated analytics to the data collected from these sensors has proven advantageous in optimization endeavors [82]. Furthermore, the application of ML algorithms, like Artificial Neural Networks (ANN), has been instrumental in optimizing a range of operations, encompassing logistics, supply chains, production, and marketing [83].

I4.0 technologies have enabled real-time monitoring and enhancements in processes [84,85,86]. Businesses and government entities aim to gain a competitive advantage by optimizing output while minimizing expenses. Enhancing productivity and quality is vital for this objective. Integrating I4.0 technologies is pivotal in achieving these goals [87] and paving the way for operational excellence [88,89,90]. Figure 6 illustrates the correlation between data and decision-making processes.

Integrating automation and IT has profoundly transformed managerial, engineering, and operational roles, making workplaces increasingly reliant on sophisticated data analysis and decision-making processes [79]. This deepened integration is pivotal in leveraging data science to convert vast data volumes into actionable insights, enhancing transparency, and improving product quality [80,81]. The application of sensors for quality control, coupled with advanced analytics applied to sensor data, supports significant optimization efforts across various sectors [82].

**Figure 7:**
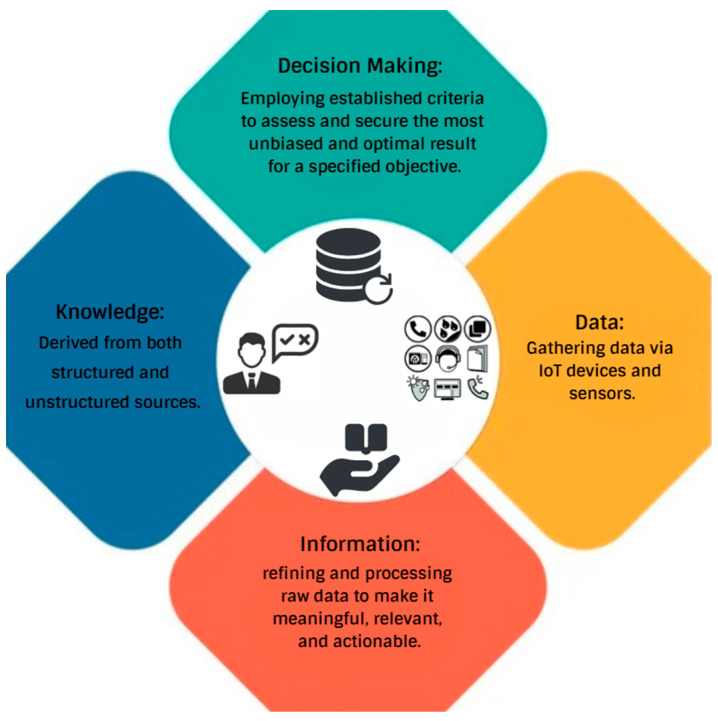
Illustrating the Connection between Data and Decision-Making Processes.

Incorporating ML algorithms such as ANN optimizes a range of operations, including logistics, supply chains, production, and marketing [83]. Real-time monitoring and process enhancements facilitated by Industry 4.0 technologies allow businesses and government entities to enhance productivity and quality, which is crucial for maintaining a competitive edge and minimizing expenses [84,85,86]. Integrating these technologies is pivotal in achieving operational excellence and continually optimizing output while minimizing expenses, as they pave the way for more innovative, more efficient operational processes [87,88,89,90]. Figure 7 illustrates the correlation between data and decision-making processes.

Our research aims to showcase how integrating computer vision technologies can revolutionize concrete structures’ traditional manual inspection processes, significantly reducing time and labor. This includes evaluating the fidelity and efficiency of well-known algorithms by benchmarking them against a custom-built CNN for detecting cracks in concrete. The potential of robotic drones in scanning bridges and transmitting images to remote servers for analysis further exemplifies the efficiency of modern inspection methods, thus minimizing resource wastage and improving the inspection process [91]. Table 3 shows some aspects of technology applications in the process of concrete inspection in bridges and their effects.

**Figure 8:**
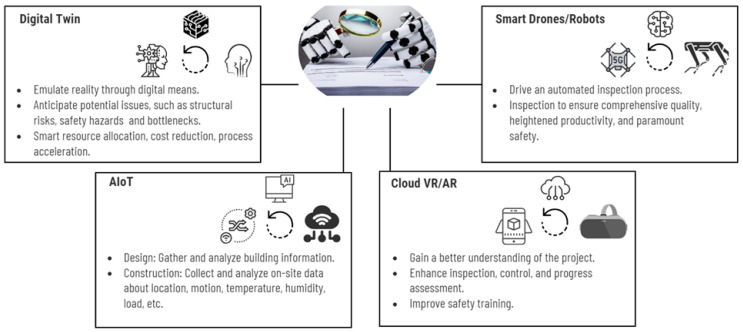
Inspection 4.0.

Integrating these advanced technologies enhances the concrete bridge inspection process, ensuring efficient, accurate, and safe inspections. Additionally, these technologies provide detailed, data-driven insights into the bridge’s condition, optimizing maintenance activities and ensuring long-term structural integrity. This integration exemplifies the application of I4.0 technologies in transforming traditional inspection processes into a more advanced, data-driven approach. Figure 8 demonstrates how these technologies collectively contribute to the modern inspection paradigm, aligned with the principles of I4.0.

This approach improves efficiency, reduces costs, ensures safety, and enables more effective decision-making based on comprehensive data analysis. The integration of Artificial Intelligence of Things (AIoT), Augmented Reality (AR), Virtual Reality (VR), and digital twins into this framework represents a significant advancement in utilizing big data and automation to support complex decision-making processes in infrastructure management [92,93,94].

## 3. Dataset

**Figure 9:**
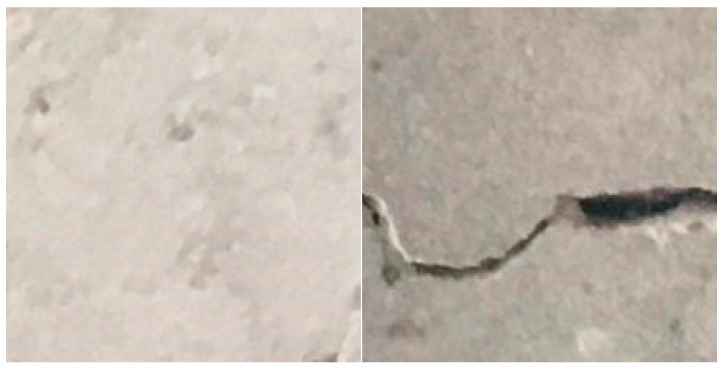
Negative (normal) and positive crack images.

The dataset’s quality and volume significantly impact the performance of DL models. To ensure optimal results, the network requires an extensive collection of images. The dataset titled Concrete Crack Images for Classification [95,96] contains concrete images with cracks. The dataset is categorized into negative (normal) and positive (cracked) images for image classification. There are 20,000 images in each category, amounting to 40,000 images, each with 227 × 227 pixel dimensions. These high-resolution images display variations in surface texture and lighting conditions. A division of 80/20 was applied for training and testing, respectively. An illustration of the images present in the dataset is shown in Figure 9.

Algorithms for classifying images typically perform better in detection tasks when they can access more images. To augment the dataset, new images were created by altering spatial characteristics, including horizontal and vertical flips, rotations, changes in image brightness, and shifts in both horizontal and vertical directions, and adjusting the magnification of existing images. DL models, with their numerous hidden neurons, depend on both the diversity and the volume [97] of the dataset utilized in training to attain high efficiency in intricate tasks [98,99]. Furthermore, data augmentation is beneficial for simulating real-world applications, as it allows capturing images from various angles and perspectives, occasionally even in inverted forms, under different conditions and using varying camera specifications.

## 4. Methodology

**Figure 10:**
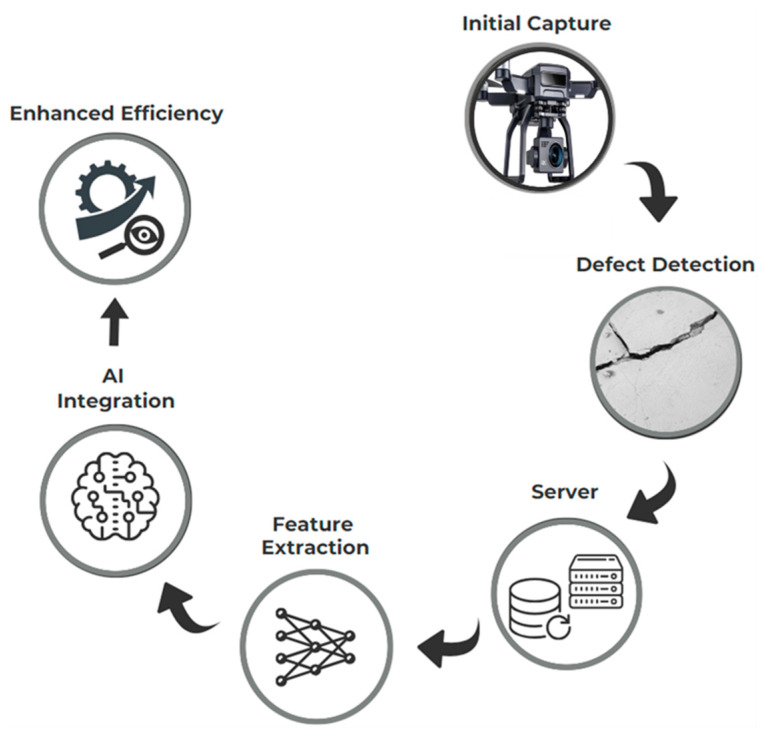
Illustration of the inspection system.

To more effectively showcase the advantages and limitations of computer-based vision. Four different ViT models were selected and benchmarked with a custom-built CNN model for this dataset. Figure 9 shows the inspection system. The camera is usually attached to a UAV, such as a drone. The UAV is being controlled remotely, and the camera takes pictures that are transmitted to the server for crack detection. In some cases, a climbing robot can do the inspection autonomously and send the images taken by the camera to the server for inspection. In all cases, Figure 10 below represents a simple system illustration.

The paper utilized the Python programming language via the Python 3.11 programming software. Python is widely used in image classification due to its rich ecosystem of libraries. These libraries offer comprehensive tools and frameworks that simplify the process of building, training, and testing image classification models. Python’s readability and simplicity enable rapid prototyping and experimentation with different architectures. Python also supports data manipulation and augmentation through libraries like NumPy and OpenCV, which are crucial for pre-processing images for classification. The final results of each coded algorithm can be measured in different ways. In our case, we relied on the confusion matrix values at the end of the testing phase and the loss and accuracy values per epoch during the training and validation phases.

### 4.1. Custom-Built CNN

**Figure 11:**
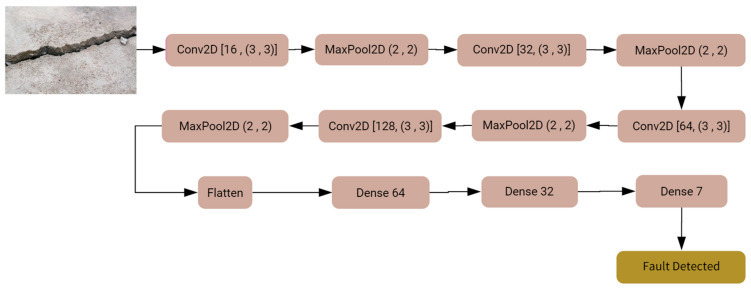
CNN model.

A custom-built CNN model tailored (see Figure 11 for an approximate illustration) for classifying colored concrete images into “images with crack” and “images without crack” integrates several components. The model often commences with an input layer designed to receive concrete image data. Given the colored nature of the images, the input dimensions typically account for the height, width, and three color channels (red, green, and blue). Following this, several convolutional layers are introduced. These layers employ filters (or kernels) to slide over the input image, detecting features by computing dot products and generating feature maps. The convolutional layers are frequently paired with activation functions like the Rectified Linear Unit (ReLU) to introduce non-linearity, enabling the model to learn intricate patterns. Pooling or subsampling layers intersperse between convolutional layers, predominantly to reduce dimensionality, focus on dominant features, and enhance computational efficiency. Max-pooling is a favored technique, where the maximum value from a group of importance in the feature map is chosen, effectively condensing the data [100,101].

**Figure 12:**
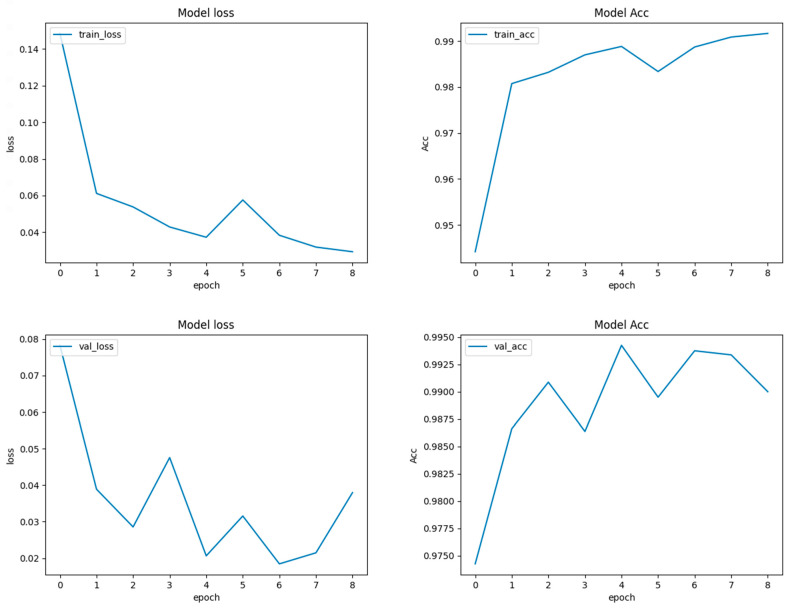
Performance of the CNN model during training and validation.

Deep CNN models usually integrate several convolutional and pooling layers in sequence, with each successive layer aiming to recognize more complex features. After these layers, the network integrates one or more fully connected (dense) layers, which interpret the recognized features and make decisions based on them. Before reaching the final classification, dropout layers might be interspersed within the fully connected layers to prevent overfitting by randomly deactivating a fraction of neurons during training [102]. Concluding the model is the output layer, which, in this case, typically consists of two neurons corresponding to the two classes: “images with crack” and “images without crack.” A softmax activation function is utilized here to output the probabilities for each class. The class with the higher probability determines the final classification. Training the model necessitates a loss function, like categorical cross-entropy for this two-class problem, and an optimizer like Adam or SGD (Stochastic Gradient Descent) to adjust weights based on the loss gradient. During the training phase, the model iteratively refines its weights by comparing its predictions to the true labels, aiming to minimize the loss and improve accuracy. The model’s effectiveness is gauged using a validation dataset, ensuring it generalizes well to unseen data. Figure 12 shows the performance of CNN during the training and validation process.

### 4.2. Visual Transformer (ViT)

ViTs are considered a significant advancement in computer vision, applying principles of transformer architecture, initially designed for Natural Language Processing (NLP) tasks, to image analysis. This approach departs from the CNNs that have traditionally dominated this domain. ViT demonstrates that transformers can achieve remarkable performance on image recognition tasks, challenging the supremacy of CNNs in computer vision and thus representing a novel approach to image classification [103,104]. The core idea behind ViT is to treat an image as a sequence of patches, akin to how a sentence is viewed as a sequence of words in NLP [105]. This methodology enables the application of transformer models directly to patches of images, allowing the model to capture complex dependencies and relationships between different parts of an image. Each image is divided into fixed-size patches, flattened, and linearly embedded. A positional encoding is added to each patch embedding.

The deployed ViT model in this paper consists of a sophisticated NN architecture that uses the ViT-B_16 configuration. The ViT-B_16 architecture represents a sizable but computationally manageable model intended for use cases where significant expressive power is needed without the full extent of resources required by the most significant transformer models. The combination of self-attention and the patch-based approach allows the ViT to learn from the local patch-level features and the global image-wide relationships, which is particularly powerful for diverse and complex image datasets.

At its core, the ViT-B_16 architecture divides an input image into fixed-size patches. In the case of the ‘B_16’ variant, this size is typically 16 × 16 pixels. These patches are treated similarly to tokens (like words in NLP) and are linearly embedded into a higher-dimensional space. The ‘B’ in the ‘ViT-B_16’ nomenclature typically stands for ‘Base’ and indicates a particular scale of the model regarding layer depth and complexity. Each embedded patch is then prepended with a learnable embedding analogous to the NLP transformers’ Classification and Sequence (CLS) token. The CLS token is a unique token used in the BERT (Bidirectional Encoder Representations from Transformers) architecture to represent the entire input sentence. It is added to the beginning of each input sentence and is used as the aggregate sequence representation for classification tasks. The final state corresponding to the CLS token is input for additional layers that make predictions. These CLS tokens will eventually hold the representation used for classification purposes. Positional embeddings are also added to retain the order information, which would otherwise be lost, as the transformer architecture does not inherently process sequential data. Once prepared, the sequence of patch embeddings is passed through a series of transformer encoder layers. These layers comprise multi-head self-attention mechanisms that allow the model to weigh the importance of different patches relative to one another. This distinctive feature of self-attention enables the model to capture global dependencies within the image. The encoder layers also contain Multilayer Perceptrons (MLPs), with each component followed by normalization steps and residual connections. In the ViT-B_16 model, the dimensionality of the MLPs’ hidden layers and the number of attention heads are more extensive than those found in more minor variants like ViT-Tiny or ViT-Small. The ‘Base’ variant balances the ‘Large’ models with even more parameters and the smaller ones that may not capture as many complex features but are faster to train. Following the transformer encoders, the representation corresponding to the CLS token embedding is passed through a final classification head, typically a superficial linear layer, to produce the output probabilities for each class. This final layer is often customized, as seen in the provided code, to match the specific number of classes in the classification task at hand.

**Figure 13:**
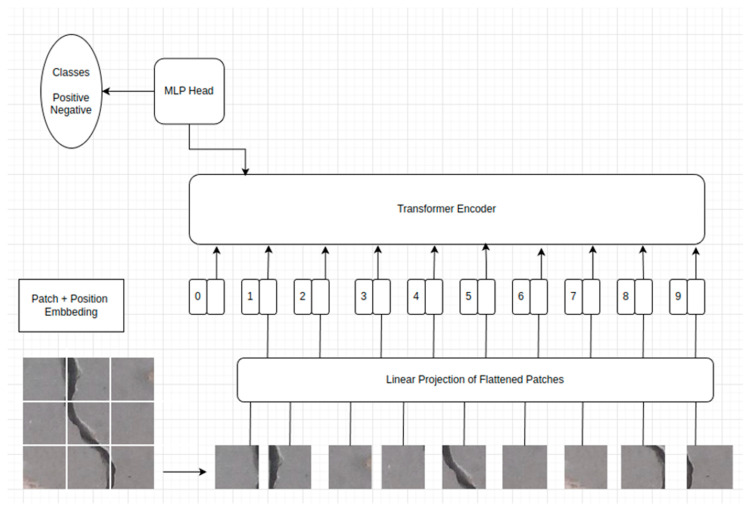
ViT model.

In adapting the model to the specific requirements of the concrete crack classification task, the original classifier head of the ViT, which determines the final output predictions, is replaced. The new classifier head is a linear layer that is sized according to the number of classes found within the training directory. This change is pivotal as it tailors the model’s output to the classification problem, allowing the model to differentiate between various types of concrete cracks. Figure 13 summarizes the ViT architecture built for concrete crack detection.

#### 4.2.1. ViT with Canny Edge Detector Enhancement

**Figure 14:**
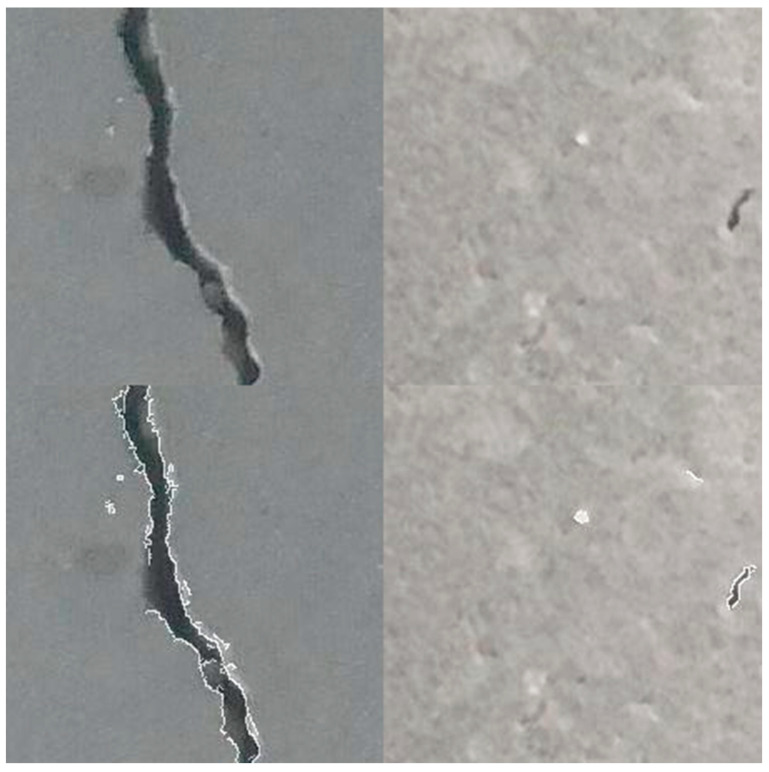
Left to right: images with cracks vs. images without cracks, and top to bottom: original images vs. enhanced images.

**Figure 15:**
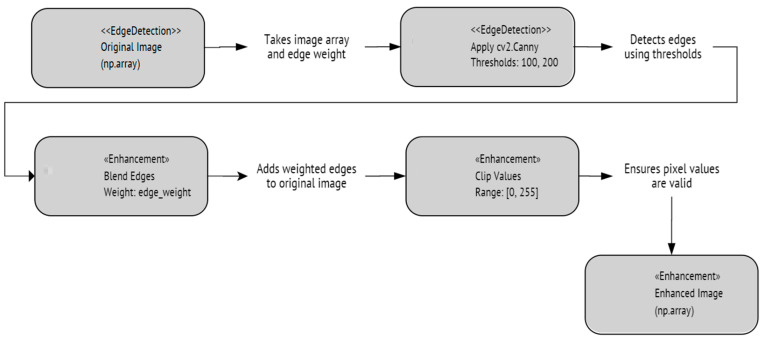
The implemented Canny edge detector algorithm.

**Figure 16:**
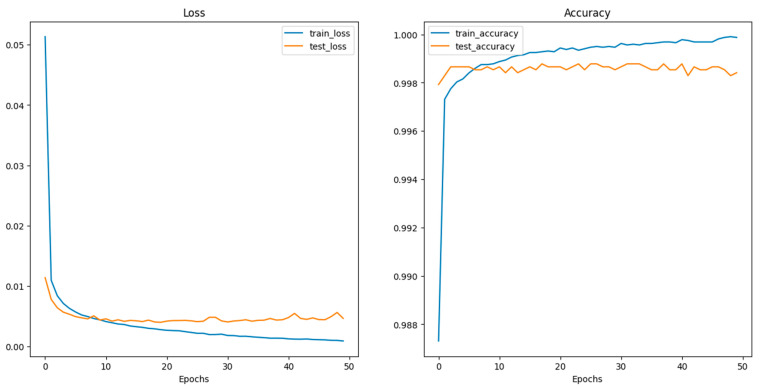
The performance of ViT with a Canny edge detector during training and validation.

The Canny edge detector is a multi-stage algorithm aiming to detect a wide range of image edges while suppressing noise. The algorithm consists of five main steps. The detector begins by applying a Gaussian blur to smooth the image, effectively reducing noise and creating a more coherent foundation for edge detection. Next, the intensity gradient of the image is computed using Sobel filters, which highlight areas of high contrast. Non-maximum suppression is applied to thin the edges, selecting only the pixels with the maximum gradient magnitude in the edge direction. This is followed by double thresholding, which categorizes pixels as strong, weak, or non-edges based on two threshold values. Finally, edges are tracked by hysteresis, where weak edges not connected to firm edges are suppressed, resulting in a refined and accurate detection of edges. This final step ensures that only meaningful edges are retained while irrelevant ones are eliminated. Figure 14 shows an illustration of the capabilities of this detector, while Figure 15 shows a flowchart of the steps being implemented in this process. Figure 16 shows the performance of ViT with a Canny edge detector during the training and validation process.

#### 4.2.2. ViT with Texture Detector Enhancement

This image enhancement process begins with grayscale conversion, where the image is converted to a single channel. This simplifies the image, making it ideal for tasks like texture analysis. Next, the grayscale image undergoes histogram equalization, which adjusts the contrast by spreading out the most frequent intensity values. This is followed by CDF (Cumulative Distribution Function) normalization, where the histogram is computed and normalized to map pixel intensities to new values, further enhancing contrast.

**Figure 17:**
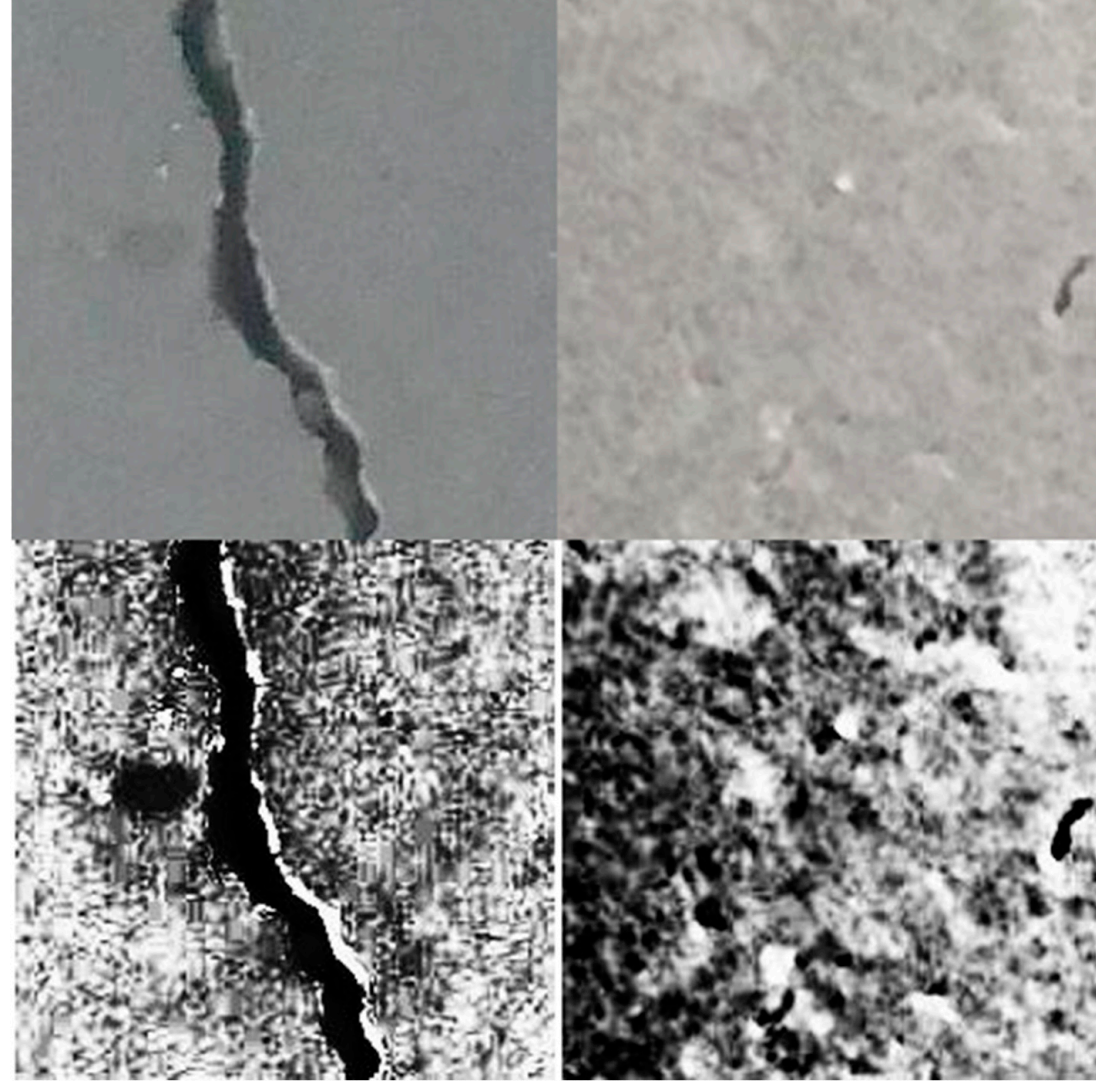
Left to right: images with cracks vs. images without cracks, and top to bottom: original images vs. enhanced images.

**Figure 18:**
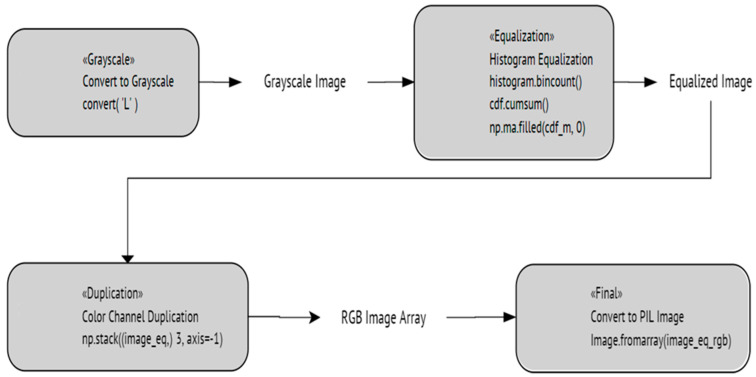
The implemented texture detector algorithm.

**Figure 19:**
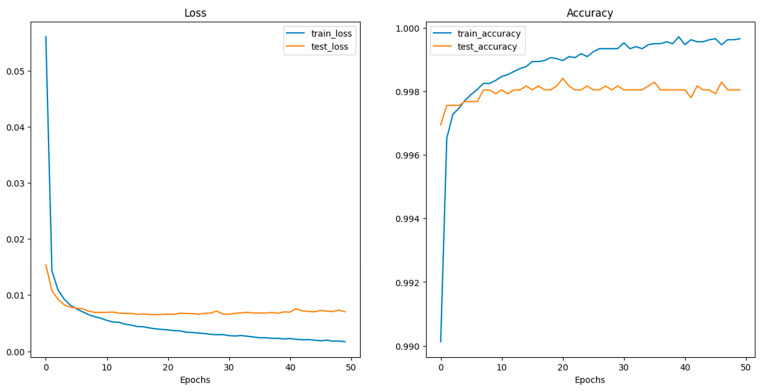
The performance of ViT with a texture detector during the training and validation process.

After histogram equalization, the single-channel image is duplicated to create a three-channel image, a necessary step for compatibility with subsequent processes that expect a three-channel format. Finally, the enhanced image is converted back to a PIL (Python Imaging Library) image from its NumPy array representation, ensuring compatibility with further processing or visualization. This final step is crucial for the image to be usable for subsequent tasks. Figure 17 shows an illustration of the capabilities of this detector. In contrast, Figure 18 shows a flowchart of the steps being implemented in this process. Figure 19 shows the performance of ViT with a texture detector during the training and validation process.

#### 4.2.3. ViT with Gaussian Blur Detector Enhancement

**Figure 20:**
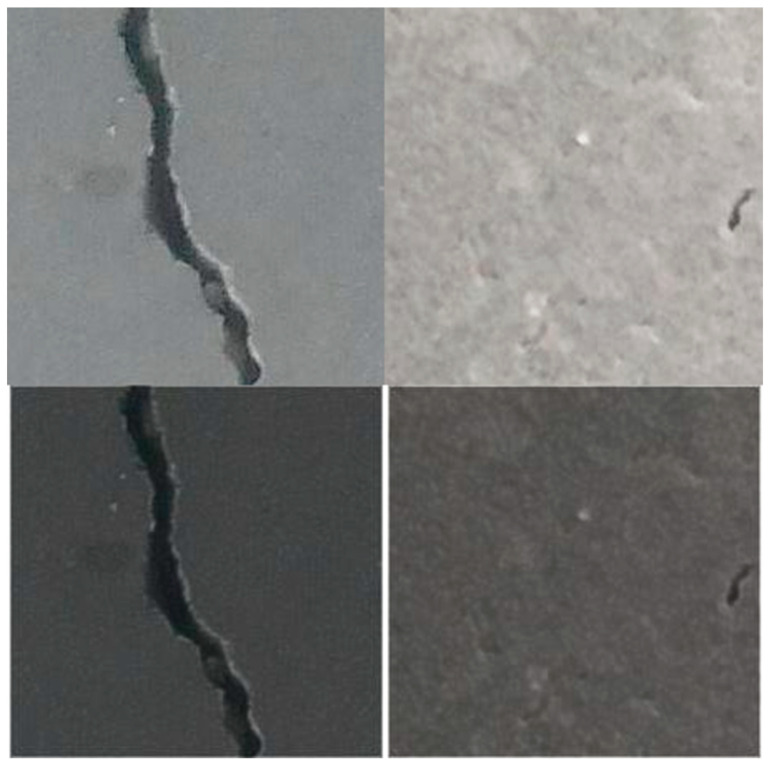
Left to right: images with cracks vs. images without cracks, and top to bottom: original images vs. enhanced images.

This image enhancement technique uses a Gaussian filter to smooth out an image, reducing noise and enhancing edges. The filter applies a mathematical formula to each pixel, averaging its value with neighboring pixels based on a Gaussian distribution. This reduces high-frequency noise, preserves low-frequency features and edges, and creates a more coherent and natural-looking image. By adjusting the radius of the Gaussian filter, the amount of blurring can be controlled, allowing for a balance between noise reduction and edge preservation. The resulting image is often more visually appealing and more accessible to analyze or process. Figure 20 shows an illustration of the capabilities of this detector.

**Figure 21:**
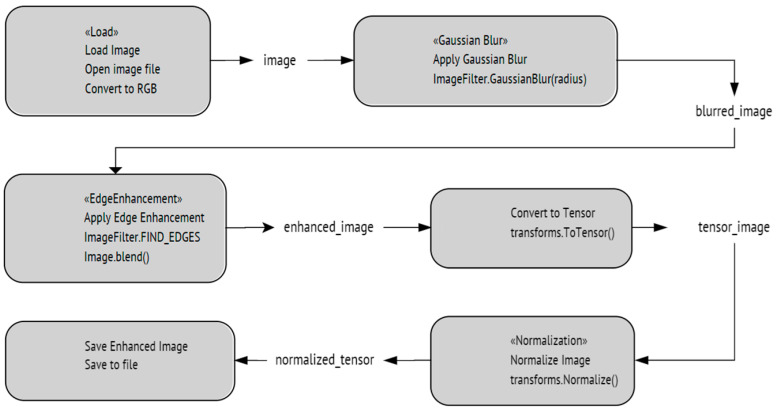
The implemented Gaussian blur detector algorithm.

**Figure 22:**
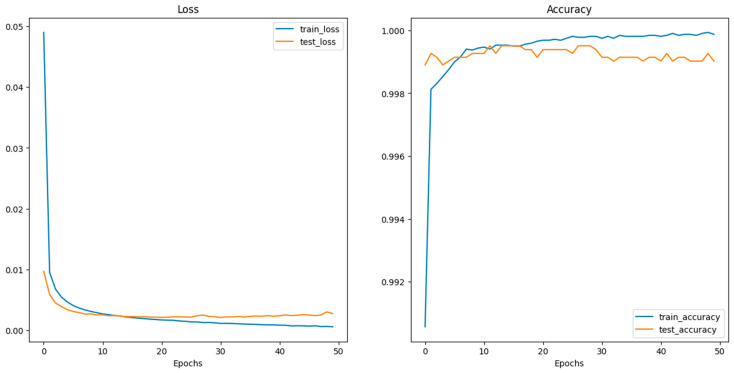
The performance of ViT with a Gaussian blue detector during the training and validation process.

In contrast, Figure 21 shows a flowchart of the steps being implemented in this process. Figure 22 shows the performance of ViT with a Gaussian blur detector during the training and validation process. Finally, the Gaussian blur detector can be summarized as follows:

- Image Capture: Feeding the image that will be processed.
- Apply Gaussian Blur: Utilize a Gaussian function to blur the image. This step involves setting the radius, or standard deviation, which determines the extent of the blur. This process smooths the image by averaging the pixels with a weighted mean, where the Gaussian function determines the weights. The critical parameter in this function is the standard deviation (σ), which controls the extent of the blurring. A larger σ results in more blurring as it increases the kernel size, effectively averaging over a larger area around each pixel. This technique helps reduce image noise and detail, which is particularly useful for pre-processing in image-processing tasks.
- Detection of Edges: Apply edge detection algorithms (like Sobel or Canny) to the blurred image to identify areas where sharp color transitions occur, indicating potential details or boundaries.
- Enhancement Decision: Analyze the detected edges to decide if further enhancement is necessary. This could involve sharpening the image or applying additional filters to enhance clarity.
- Final Adjustment: Make final adjustments to the image contrast, brightness, or other attributes to ensure optimal visibility of essential features.

#### 4.2.4. ViT with Local Binary Patterns (LBP) Detector Enhancement

**Figure 23:**
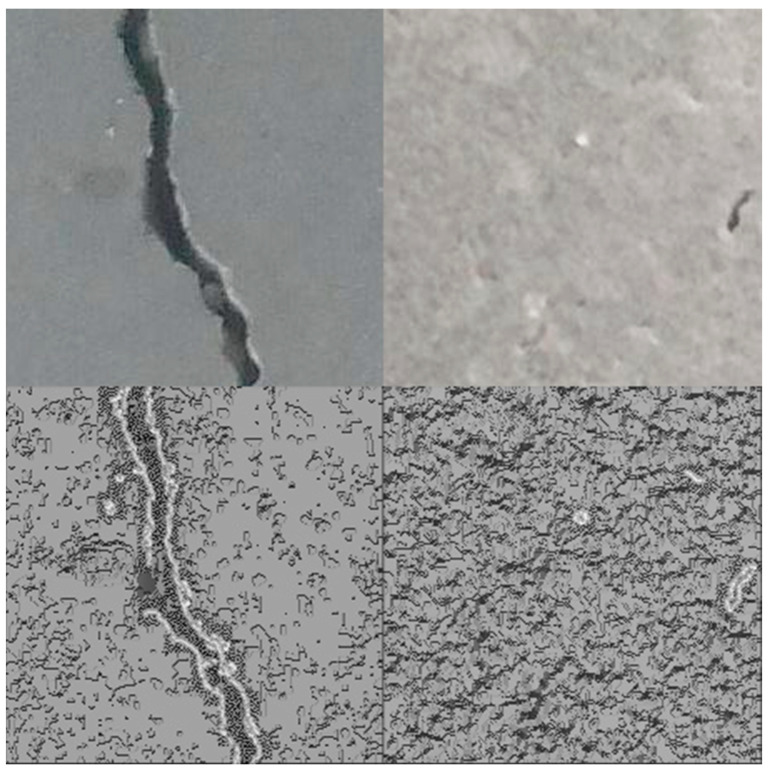
Left to right: images with cracks vs. images without cracks, and top to bottom: original images vs. enhanced images.

**Figure 24:**
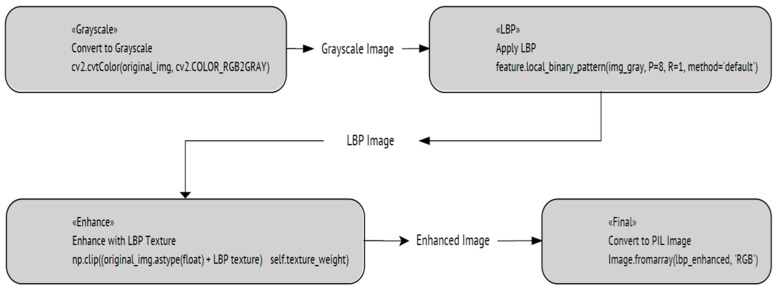
The implemented LBP detector algorithm.

**Figure 25:**
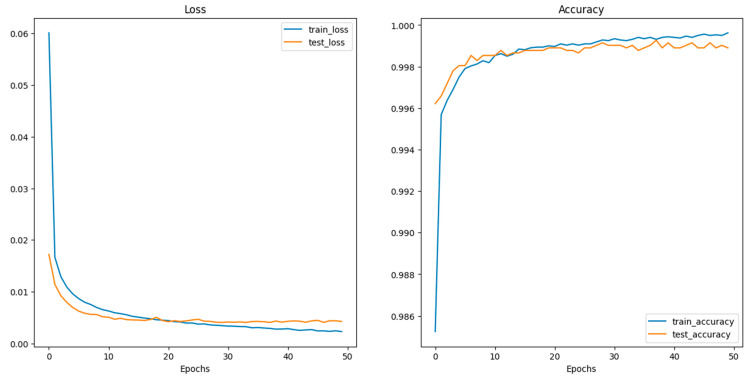
The performance of ViT with LBP during the training and validation process.

LBP is a powerful method used for texture classification. It compares each pixel with its surrounding neighbors. It encodes this relation into a binary number, effectively capturing the local texture information. After computing the LBP, it is converted into an 8-bit unsigned integer format (uint8), ensuring compatibility with other image processing functions and avoiding issues with data types that can arise during mathematical operations on images. The key to enhancing the original image is to blend it with the textured information obtained from the LBP. This blending is controlled by a parameter, which defines how strongly the texture features should influence the final image. The result is an image that retains its original content but has an emphasized texture pattern, which can be crucial for task recognition or feature detection. Figure 23 shows an illustration of the capabilities of this detector. In contrast, Figure 24 shows a flowchart of the steps being implemented in this process. Figure 25 shows the performance of ViT with LBP during the training and validation process.

## 5. Results and Discussion

The effectiveness of the image processing algorithm is often determined by the components of its confusion matrix, which consists of True Positive (TP), False Positive (FP), False Negative (FN), and True Negative (TN). The confusion matrix for each of the five models is depicted in Table 4.

Table 4 presents the confusion matrix values for different models, evaluating performance for both the positive and negative classes. Each model, including a custom CNN, ViT with Canny, texture, Gaussian, and LBP detectors, is assessed by FP, FN, TP, and TN metrics. The custom CNN shows a balanced detection capability across both classes. At the same time, the ViT variations display varied effectiveness, with the Gaussian Detector and LBP Detector showing notable precision in correctly identifying TPs and TNs. Several conclusions regarding the models can be drawn from Table 4, including:

- Custom CNN: This model has a higher count of FP and FN in the negative class than the positive, indicating it may slightly favor the positive class in classification accuracy.
- ViT with Canny Detector: This shows a more balanced performance but slightly better accuracy in detecting the positive class, as indicated by lower FP and FN.
- ViT with Texture Detector: This model displays identical performance across both classes, with equal numbers for all metrics, suggesting a balanced but potentially less discriminative ability.
- ViT with Gaussian Detector: It exhibits high accuracy, particularly in identifying the positive class with very low FP and FN, highlighting its effectiveness in precise classifications.
- ViT with LBP Detector: Similar to the Gaussian model, it shows high accuracy and a low misclassification rate, particularly in the positive class.

In classification models, accuracy (TP + TN/TP + TN + FP + FN) is a crucial metric representing the proportion of correct predictions. This is particularly vital in applications like concrete crack detection for bridges, where it influences maintenance decisions and safety measures. Precision (TP/TP + FP), another key metric, measures the ratio of TP to all positive predictions, which is crucial for optimizing resources and minimizing unnecessary actions. Recall, or sensitivity (TP/TP + FN), ensures that actual defects are identified, enhancing safety and preventive maintenance. The F-measure combines precision and recall to provide a balanced performance assessment (2 × precision × recall/precision + recall). Specificity (TN/TN + FP), or the TN rate, helps avoid misidentifying healthy structures as damaged, optimizing maintenance efforts and resource allocation. G-means integrate sensitivity with specificity and precision with recall, providing a holistic evaluation of a model’s performance, which is especially important in imbalanced datasets. These metrics collectively ensure crack detection systems’ effectiveness, efficiency, and reliability in bridge maintenance. Table 5 summarizes the benefits of using each performance metric in concrete crack detection. In Table 6, the average values for accuracy, precision, sensitivity, specificity, G-mean1, G-mean2, and F1 scores across both classes of concrete images are presented for all employed models. Moreover, Table 7 displays the number of epochs required to train each model to attain the minimum loss and maximum accuracy and the corresponding time taken.

Examination of Table 6 reveals nearly identical performance measurements across various image classification models, a phenomenon possibly linked to factors like dataset characteristics and performance saturation. Furthermore, the table reveals that ViT models combined with image enhancement algorithms outperformed the CNN model. The dataset’s features, such as size, complexity, and noise levels, could have influenced the performance of the CNN model. The ViT with Gaussian detector algorithm has achieved the best results out of all models on all the metrics of performance measurements. The primary objective of this paper was to assess the accuracy and efficiency of various DL image-processing algorithms for detecting cracks in concrete. Most models demonstrated exceptionally high-performance metrics, averaging around 99.8%. High-sensitivity tests yield positive results for detecting damaged concrete in diagnostic and inspection scenarios.

In contrast, high-specificity tests yield negative results for normal concrete. Consequently, it is crucial to consider sensitivity and specificity to understand any inspection test comprehensively. The G-mean functions provide a balanced metric, considering sensitivity and specificity as well as sensitivity and precision. All the examined models consistently exhibited G-mean values of at least 99.1%, affirming their suitability for early detection of concrete cracks. Furthermore, the results can be summarized below as follows:

- CNN: This model displays robust overall metrics, indicating a well-balanced approach to accurately classifying and rejecting images. It is slightly lower in performance than some ViT models, likely due to its simpler or less specialized architecture. CNNs may lack specific optimizations that specialized detectors incorporate, leading to slightly lower performance metrics than more tailored solutions.
- ViT with Canny Detector: The Canny edge detector enhances feature definition by focusing on edges, which is crucial for image classification tasks. This sharpens the model’s ability to discern distinct features, improving precision and specificity by reducing false positives. It has exceptionally high scores in precision and specificity, reaching 100%, suggesting this model excels at minimizing false positives, likely due to the Canny detector’s edge-detection capabilities enhancing the model’s ability to discern features more sharply.
- ViT with Texture Detector: This model likely uses texture-based features for classification, providing a uniform approach across different metrics. However, it might not excel without additional context or detail refinement from other specialized detectors. The uniformity across all metrics suggests that this model is highly consistent.
- ViT with Gaussian Detector: Incorporating Gaussian blur helps in smoothing out noise and variations within the images, enhancing the TP rate (sensitivity). This process might improve the model’s identification of relevant features by minimizing background noise interference. Leading in the F-measure ensures that this model is particularly effective in identifying TP.
- ViT with LBP Detector: Local Binary Patterns are effective for texture classification, which can enhance sensitivity. However, the method might also introduce noise in the form of false positives if the texture patterns are not distinct enough between classes, slightly reducing precision and specificity.

The variations in performance can be attributed to how each model and its associated techniques handle image features differently, their sensitivity to image alterations, and their inherent design geared towards optimizing specific aspects of image classification. Each detector’s unique approach to processing visual information results in these observable differences in performance metrics.

An epoch in the context of ML, particularly in training NN, denotes a complete iteration through the dataset during the training phase. In other words, an epoch is completed when the model has processed every sample in the training dataset once. During an epoch, the neural network’s weights are adjusted to minimize the error or loss function as it learns from the dataset. The training process usually comprises multiple epochs to ensure effective learning of the underlying patterns and relationships in the data. The number of epochs is a hyperparameter, allowing adjustment based on the specific problem and the desired model performance. Based on the values recorded in Table 7, it becomes clear that although the CNN model had the lowest performance, it also had the shortest running time without compromising so much of its performance. Various image classification models’ training times and efficiencies reflect the complexities inherent to their specific computational processes. With only nine epochs, the Custom CNN showed the quickest training cycle despite a high average time per epoch, completing its training in 1710 s. This contrasted with the ViT models, which required 50 epochs each. The ViT with Canny and the ViT with Texture models had relatively lower per-epoch times, leading to total times of 7234 s and 6986 s, respectively, indicating efficiency in processing.

Meanwhile, the ViT with Gaussian exhibited a moderate epoch duration but an overall longer training time due to the computational demands of Gaussian blurring. The most time-intensive model, the ViT with LBP, had significantly longer epoch times, culminating in a total training time of 15,609 s, reflecting the high computational load required for detailed texture analysis through LBP. Each model’s training duration underscores the trade-offs between computational complexity and processing efficiency in handling different aspects of image analysis.

## 6. Limitations and Future Works

In discussing the limitations of image classification algorithms for detecting concrete cracks, it is crucial to consider several key factors affecting their performance and applicability. The quality of input data is paramount, as poor lighting, low resolution, or noisy images can significantly hinder the algorithm’s ability to detect cracks accurately. Concrete cracks vary in forms like spalling, delamination, and cracking, each with distinct characteristics, often requiring multiple or fine-tuned models for effective detection. Environmental changes such as lighting, shadows, and weather conditions can also alter crack appearances, complicating consistent performance. Additionally, concrete surfaces’ heterogeneous and textured nature can make it difficult to distinguish actual cracks from natural variations.

Dataset imbalances, where the data may predominantly consist of images of healthy concrete, can lead to biased models that underperform in detecting actual cracks. Overfitting presents another challenge, as algorithms overly trained on specific datasets may not generalize well to new, unseen data. The high computational demands of advanced image classification models, especially those based on deep learning, may not be practical for real-time or on-site applications where quick decision-making is essential. Moreover, many deep learning algorithms operate as “black boxes” with limited interpretability, which can be problematic in critical infrastructure applications.

Cybersecurity is a vital consideration, as using digital systems, IoT devices, and cloud services in infrastructure monitoring exposes these systems to potential cyber threats, risking the safety and integrity of the infrastructure [106]. Addressing these limitations requires enhancing data quality, diversifying training datasets, developing robust models against environmental variations, and securing digital infrastructures to improve the reliability and effectiveness of image classification systems in concrete crack detection.

The specific methods used in this study, such as ViT combined with image enhancement detectors like Canny, texture, Gaussian, and LBP, exhibit inherent limitations. While these models show high accuracy on the dataset, their ability to generalize to new, unseen datasets or real-world scenarios with varying conditions may be limited. Additionally, the computational intensity required for processing and the potential for overfitting pose significant challenges. These issues underscore the need for further research to enhance the robustness and applicability of these algorithms in diverse and practical settings.

## 7. Conclusions

One in every nine bridges is expected to collapse across the United States. Therefore, AI-based applications using machine vision can be designed to assist in detecting damaged concrete in bridge structures. This paper showed how computer vision inspection via ViTs, alongside diverse image enhancement detectors like Canny, texture, Gaussian, and LBP, significantly improved concrete crack detection. These technological integrations refine the precision of crack detection and establish a new benchmark by comparing multiple state-of-the-art computer vision models on a consistent dataset, ensuring a comprehensive evaluation of their performance. While ViT models demonstrate nearly perfect accuracy, the deployed CNN model stands out for its remarkable performance by requiring significantly less training time than the ViTs while delivering comparable results, showcasing its efficiency in enhancing safety through reliable detection and timely maintenance.

Additionally, this approach aligns with I4.0 objectives by automating and optimizing the resource-intensive process of manual inspections, reducing operational costs, and facilitating more efficient maintenance schedules. The paper also detailed performance metrics nearing 100% utilizing ViT combined with diverse image enhancement detectors, showcasing the prowess of computer vision in bridge inspection. This underscores the transformative potential of advanced computer vision techniques, particularly the efficiency of custom CNNs, in enhancing concrete inspection processes and setting a new standard for precision and reliability in infrastructure maintenance. Future research should prioritize leveraging the efficiency and accuracy of custom CNNs to address remaining challenges in automated crack detection, integrating image classification with non-destructive evaluation methods for enhanced accuracy, and enabling real-time crack monitoring for prompt maintenance. Advanced cybersecurity is also needed to protect digital infrastructure management systems, improve data quality and imaging for more precise crack detection, and apply transfer learning to adapt algorithms to specific domains. Additionally, integrating drones and robotics for efficient inspections and developing explainable AI for transparent crack detection processes are pivotal.

A future direction for research might include the experimental testing of crack detection. Initially, a comprehensive dataset of images or videos showcasing various surface cracks is collected under different environmental conditions to ensure diversity. These images are then pre-processed to enhance visual quality, utilizing noise reduction and contrast enhancement techniques. The selected computer vision algorithms, like edge detection or deep learning models (e.g., CNNs), are applied to detect and categorize cracks. Performance is assessed by comparing the algorithm’s results with manually annotated ground truth data using metrics like precision and recall. Finally, the system is tested in real-world settings to evaluate its practical effectiveness and robustness. It is followed by iterative adjustments based on feedback to enhance its accuracy and adaptability. Finally, assessing crack depth remains a critical area lacking a dedicated DL solution. Table 8 shows some areas where future research direction might be needed.

## Figures and Tables

**Table 1:** Areas of Improvement in the Bridge Inspection Process [2].

Area	Improvement
Changes to inspection interval determination	A scoring system for determining inspection intervals for fractured-critical bridges was proposed by Parr et al. [3]. In addition, a probabilistic framework to create risk-based inspection intervals was built using the event-tree model and presented by Orcesi and Frangopol [4]. Also, Washer et al. [5] proposed deriving inspection intervals from a risk-based approach instead of a time interval using an expert panel to assess and predict the condition and failure of bridge elements.
Utilizing decision-making tools	Creating technology-based decision support programs in selecting appropriate remedial activities and allocating resources for bridge maintenance programs [6]. Thus, more bridges can be scanned [7].
Deployment of technology	Recently, the bridge inspection process started to deploy Industry 4.0 technologies [8], such as drones [9,10], photogrammetry [11,12,13], virtual reality [12,14], and database management systems.
Reliability	Establishing a time-dependent reliability analysis that can predict future structural performance using information collected from past visual inspections [15]. The American Association of State Highway and Transportation Officials (AASHTO) has addressed the standardization of bridge inspections. The AASHTO released the Manual for Bridge Element Inspection (MBEI) in 2013 and updated it in 2019. It guides bridge element-level assessment with definitions for the condition and number of elements. It codifies possible responses to element conditions [16].

**Table 2:** The importance of bridge inspections.

Area	Key Reason
Public safety [17]	Regular bridge inspections help identify potential safety hazards or structural issues that could lead to accidents or collapse. Ensuring the safety of all bridge users, including pedestrians, cyclists, and motorists, is a top priority.
Structural integrity [18]	Inspections allow engineers to assess the bridge’s structural condition and detect any signs of deterioration, corrosion, or damage. Early detection of structural issues can prevent catastrophic failures and allow for timely repairs.
Maintenance and repair [19]	Bridge inspections help identify areas that require maintenance or repairs, allowing authorities to address problems before they become critical. Regular maintenance can help avoid costly and time-consuming repairs or replacements.
Lifespan extension [20]	Through regular inspections and proper maintenance, the overall lifespan of a bridge can be prolonged. This helps maximize the return on investment for public infrastructure projects and ensures that bridges continue to serve their intended purpose for an extended period.
Resource allocation [21]	By conducting routine bridge inspections, authorities can prioritize maintenance and repair projects based on the severity of the issues identified. This facilitates the efficient allocation of resources to the most critical areas, ensuring that funds are used effectively to maintain and improve infrastructure.
Regulatory compliance [22]	In many countries, government regulations mandate bridge inspections to ensure all bridges meet safety and structural standards. Regular inspections help to ensure compliance with these regulations and reduce the risk of legal and financial penalties.
Environmental factors [23]	Inspections can help identify the impact of environmental factors, such as erosion, flooding, or temperature fluctuations, on the bridge’s structural integrity. This information can be used to plan for future maintenance or improvements to mitigate the effects of these factors.

**Table 3:** List of I4.0 technologies and their effects on the concrete inspection process in bridges.

Technologies	Effect
Computer vision technology	Computer-based vision technology can be used to automate the process of detecting defects, cracks, or other signs of damage in concrete structures. Advanced image processing algorithms can analyze high-resolution images captured by cameras, drones, or other devices to identify areas of concern. Computer vision technology can be used to inspect hard-to-reach or hazardous areas of the bridge, reducing the need for inspectors to work in dangerous conditions. This improves safety for inspection teams and allows for more thorough inspections in regions that might otherwise be challenging to access.
Data-driven decision making	Integrating computer vision technology into the inspection workflow allows teams to rapidly and precisely collect and interpret vast amounts of data. Such an approach, centered on data, enhances decision-making in areas like maintenance, repair, and the distribution of resources, leading to more effective bridge management. The data collected by robotics, drones, and remote sensing technologies can be analyzed using advanced software and machine learning algorithms to detect patterns, trends, and anomalies. This helps inspectors make data-driven decisions and prioritize maintenance activities based on the severity of the issues identified.
Real-time monitoring	By integrating computer vision technology with IoT devices, it is possible to establish a real-time monitoring system for bridge structures. This can provide early warnings of potential structural issues, allowing authorities to take proactive measures to maintain safety and structural integrity.
Robotics	Robotic systems can access hard-to-reach or hazardous areas of the bridge, reducing the need for manual inspections in these locations. Robotics equipped with sensors, cameras, and advanced imaging technologies can collect high-resolution images and data for detailed analysis. Some robots can also perform tasks such as cleaning or applying sealants to cracks, improving maintenance efficiency.
Drones	Unmanned Aerial Vehicles (UAVs), or drones, can inspect the bridge, capturing high-resolution images and videos from various angles. Drones allow inspectors to quickly assess the overall condition of the bridge, identify defects or damage, and access difficult-to-reach areas with minimal risk to personnel. Additionally, drones can be equipped with advanced sensors, such as Light Detection and Ranging (LiDAR), to collect more detailed structural data.
Remote sensing	Remote sensing technologies, such as LiDAR and Ground-Penetrating Radar (GPR), can gather detailed information about the bridge’s structural components, including detecting internal defects, corrosion, and other issues not visible to the naked eye. Consequently, these technologies offer a more detailed insight into the bridge’s status, facilitating improved decisions regarding maintenance, repairs, and the distribution of resources.

**Table 4:** Values of the confusion matrix for each model for both positive and negative classes.

Custom CNN				
	FP	FN	TP	TN
Negative	71	6	3994	3929
Positive	6	71	3929	3994
ViT with a Canny Detector				
	FP	FN	TP	TN
Negative	5	8	3992	3995
Positive	8	5	3995	3992
ViT with a Texture Detector				
	FP	FN	TP	TN
Negative	8	8	3992	3992
Positive	8	8	3992	3992
ViT with a Gaussian Detector				
	FP	FN	TP	TN
Negative	5	3	3997	3995
Positive	3	5	3995	3997
ViT with an LBP Detector				
	FP	FN	TP	TN
Negative	6	3	3997	3994
Positive	3	6	3994	3997

**Table 5:** Performance metrics used in concrete crack detection.

	Accuracy	Precision	Recall	F-Measure	Specificity	G-Mean1	G-Mean2
Safety	x	x	x				
Cost reduction	x		x				
Efficiency	x			x			
Longevity	x						
Resource allocation		x			x		
Minimizing false alarms		x			x		
Maintenance		x	x	x			
Increase reliability		x	x	x	x	x	x
More balanced assessment				x		x	x
Robustness to imbalanced data				x		x	x
Optimization						x	x

**Table 6:** Average values for accuracy, precision, sensitivity, specificity, G-mean1, G-mean2, and F1 scores.

Model	Accuracy	Precision	Sensitivity	F1-Score	Specificity	G-Mean2	G-Mean1
Custom CNN	99.04%	99.05%	99.04%	99.038%	99.04%	99.03%	99.05%
**ViT with**							
Canny Detector	99.83%	99.87%	99.80%	99.84%	99.87%	99.84%	99.84%
Texture Detector	99.80%	99.80%	99.80%	99.80%	99.80%	99.80%	99.80%
Gaussian Detector	99.90%	99.87%	99.92%	99.90%	99.87%	99.90%	99.90%
LBP Detector	99.89%	99.85%	99.92%	99.89%	99.85%	99.89%	99.89%

**Table 7:** Number of epochs and time taken for each model.

Model	Epochs	Average Time Per Epoch (s)	Total Time (s)
Custom CNN	9	190	1710
ViT with Canny	50	144.68	7234
ViT with Texture	50	139.72	6986
ViT with Gaussian	50	149	7450
Vit with LBP	50	312.18	15,609

**Table 8:** Future research directions.

Direction	Areas
Developing new algorithms	Researchers can focus on designing more sophisticated algorithms tailored explicitly for concrete fault detection, incorporating domain-specific knowledge and expertise. Concentrating on the depth of the neural model plays a pivotal role in enhancing efficiency while conserving computational resources while identifying cracks. Other methods, such as attention gates, can be integrated with CNN to increase pixel-level accuracy, which requires attention as well.
Integration with other non-destructive inspection methods	Creating a multi-modal system by combining image classification algorithms with other non-destructive evaluation techniques, such as ground-penetrating radar, ultrasonic testing, or infrared thermography, can improve the fault detection process and overall accuracy.
Real-time monitoring and fault detection	Develop systems capable of real-time monitoring and fault detection, enabling prompt identification and repair of defects, thus extending the service life of bridges and reducing maintenance costs.
Enhancing cybersecurity	As digital systems become more integrated into infrastructure management, ensuring the security and privacy of these systems will be crucial. Future research could focus on developing advanced cybersecurity measures to protect against threats and vulnerabilities.
Improving data quality	Investigate methods to enhance the quality of images used for fault detection, such as advanced image preprocessing techniques, image enhancement, or higher-resolution imaging sensors. Furthermore, there is a need for a uniform dataset to assess network designs and associated operations. The skewed nature of unbalanced datasets can compromise network efficiency, necessitating effective strategies to address these challenges.
Transfer learning and domain adaptation	Study the application of transfer learning and domain adaptation techniques to improve the performance of image classification algorithms when applied to concrete fault detection in bridges, especially in cases where labeled data is scarce.
Explainable AI	Develop more transparent and interpretable image classification algorithms, enabling engineers and decision-makers to better understand the underlying reasons for fault detection results and build trust in the system. Furthermore, the complex parameterization of DL models demands significant memory and rapid computational capabilities, making their practical deployment a subject of ongoing investigation.
Integration of Industry 5.0	Investigate the potential of further integrating collaborative robots, advanced NLP engines (ChatGPT, for example), Digital Triplet, and AIoT and their effect on maintaining infrastructures and their impact on the overall sustainability of infrastructures, considering aspects such as resource consumption, environmental impact, and long-term maintenance costs.
Crack depth	Assessing the depth of a crack can provide insights into its seriousness, yet there is not a recognized DL method specifically designed for this purpose.

## Data Availability

The data presented in this study are available on request from the corresponding author.

## Abbreviations

Industry 4.0 (I4.0)
Artificial Intelligence (AI)
Machine Learning (ML)
American Society of Civil Engineering (ASCE)
Value Stream Mapping (VSM)
American Association of State Highway and Transportation Officials (AASHTO)
Manual for Bridge Element Inspection (MBEI)
Structural Health Monitoring (SHM)
Deep Learning (DL)
Neural Networks (NN)
Convolutional Neural Networks (CNN)
Deep Belief Networks (DBN)
Long Short-Term Memory (LSTM)
Recurrent Neural Network (RNN)
Fully Convolutional Neural Networks (FCN)
Region Proposed Networks (RPN)
Deep Convolutional Neural Networks (DCNN)
Visual Transformer (ViT)
Information Technology (IT)
Artificial Neural Networks (ANN)
Unmanned Aerial Vehicle (UAV)
Light Detection and Ranging (LiDAR)
Ground-Penetrating Radar (GPR)
Artificial Intelligence of Things (AIoT)
Augmented Reality (AR)
Virtual Reality (VR)
Rectified Linear Unit (ReLU)
Stochastic Gradient Descent (SGD)
Natural Language Processing (NLP)
Classification and Sequence (CLS)
Bidirectional Encoder Representations from Transformers (BERT)
Multilayer Perceptrons (MLP)
Cumulative Distribution Function (CDF)
Python Imaging Library (PIL)
Local Binary Patterns (LBP)
True Positive (TP)
True Negative (TN)
False Positive (FP)
False Negative (FN)
